# Impact of COVID-19 on outcomes with teclistamab in patients with relapsed/refractory multiple myeloma in the phase 1/2 MajesTEC-1 study

**DOI:** 10.1038/s41408-024-01160-1

**Published:** 2024-10-21

**Authors:** Niels W. C. J. van de Donk, Nizar Bahlis, Luciano J. Costa, María-Victoria Mateos, Ajay K. Nooka, Aurore Perrot, Alfred L. Garfall, Pragya Thaman, Keqin Qi, Clarissa Uhlar, Katherine Chastain, Margaret Doyle, Saad Z. Usmani

**Affiliations:** 1 Amsterdam University Medical Center, Vrije Universiteit Amsterdam, Amsterdam, The Netherlands; 2 Arnie Charbonneau Cancer Institute, University of Calgary, Calgary, AB Canada; 3 University of Alabama at Birmingham, Birmingham, AL USA; 4 University Hospital of Salamanca/IBSAL/CIC/CIBERONC, Salamanca, Spain; 5 Winship Cancer Institute, Emory University, Atlanta, GA USA; 6 Centre Hospitalier Universitaire de Toulouse, Institut Universitaire du Cancer de Toulouse-Oncopole, Toulouse, France; 7 Abramson Cancer Center, Perelman School of Medicine, University of Pennsylvania, Philadelphia, PA USA; 8 Janssen Research & Development, Bridgewater, NJ USA; 9 Janssen Research & Development, Titusville, NJ USA; 10 Janssen Research & Development, Spring House, PA USA; 11 Janssen Research & Development, Raritan, NJ USA; 12 Janssen Sciences Ireland, Dublin, Ireland; 13 Memorial Sloan Kettering Cancer Center, New York, NY USA

**Keywords:** Myeloma, Molecularly targeted therapy

Dear Editor,

The risk of infection in patients with multiple myeloma (MM) is high, particularly for those with relapsed/refractory MM (RRMM), who typically exhibit substantial immune dysfunction due to multiple prior therapies as well as MM itself [1, 2]. The recent COVID-19 pandemic had an impact on patients with MM compared with non-MM patients, including a higher risk of infection, a higher excess mortality rate, and decreased survival in 2020 compared with 2019 [3]. Patients with MM are now known to be particularly vulnerable to COVID-19 infection [4]; COVID-19 mortality rates of up to 57% have been reported in patients with MM across different institutions [5], with hematologic cancers associated with higher infection severity and mortality than other tumor types [6].

At the onset of the pandemic, hospitals and health systems globally were immediately placed under unprecedented pressure. There were no approved COVID-19 treatments or vaccines and no clear strategy or unified approach to managing MM in the setting of COVID-19 infection, where lockdowns necessitated changes in normal treatment approaches such as the introduction of remote consultations and potential delays in MM therapy [7]. This created an extraordinary set of circumstances for healthcare professionals and patients involved in clinical studies at that time.

Recruitment for the phase 1/2 MajesTEC-1 study evaluating teclistamab—the first approved B-cell maturation antigen (BCMA) × CD3 bispecific antibody for the treatment of triple-class exposed RRMM—ran concurrently with the start of the COVID-19 pandemic. The majority of the 165 patients in the teclistamab recommended phase 2 dose (RP2D) cohort were enrolled between March 2020 and March 2021 (*n* = 151; 91.5%), overlapping with peak infection and death rates worldwide [8]. Given the novel nature of BCMA-directed bispecific antibodies at the time, this was the first study informing the MM-treating community on their infection profile (including the risk of COVID-19). Infection management during MajesTEC-1 was based on existing institutional protocols, across which implementation of preventive strategies varied widely [9]. Furthermore, depending on country, the first vaccines were not available until the end of 2020 and early 2021 [10]—at least 9 months into study enrollment; similarly, effective COVID-19 antivirals were not available outside of a hospital setting until late 2021 [11]. This is particularly significant because the characteristics of the MajesTEC-1 population overlapped with many of the features subsequently identified as risk factors for COVID-19 morbidity and mortality in patients with MM, such as age, suboptimal disease control, immunosuppression, and certain comorbidities [4, 5]. Although access to routine healthcare was severely disrupted during the pandemic, continuing active and efficacious treatment was critical in maintaining disease control [4, 5], particularly for the heavily pretreated, highly refractory MajesTEC-1 population for whom outcomes with standard treatments were poor and options for long-term control of MM were extremely limited [12].

In the context of the unique clinical situation at the time of study enrollment, we undertook a post hoc analysis of MajesTEC-1 to evaluate the potential impact of COVID-19 on patient outcomes with teclistamab.

Full details of MajesTEC-1 (NCT03145181/NCT04557098) have been published previously [12]. Per protocol, precautions to mitigate infection risk were recommended and evolved with additional data and clinical experience over time, with infections (including COVID-19) managed per institutional guidelines (e.g., use of immunoglobulin replacement) and/or teclistamab interruption [9]. Annual inactivated influenza and COVID‐19 vaccinations (including booster doses) were recommended when available [9]. All patients treated with teclistamab in the RP2D cohort (*N* = 165) were included in the overall study analysis (data cut-off: January 4, 2023; median follow-up: 22.8 months). In this post hoc analysis, patients with grade 5 COVID-19 infection were censored at the time of last disease evaluation for analyses of progression-free survival (PFS; *n* = 17) and duration of response (DOR; *n* = 13) if the death occurred without disease progression. For overall survival (OS), all patients who died from COVID-19 were censored at the time of death (*n* = 18). Outcomes were analyzed overall, in patients with complete response or better, by number of prior lines of therapy (≤3 or >3), and in the phase 2 efficacy population (including 110 patients enrolled on or before March 18, 2021).

The MajesTEC-1 population of 165 patients had a median age of 64 years (range, 33–84) and had previously received a median of five prior lines of therapy (range, 2–14); 128 (77.6%) were triple-class refractory and 50 (30.3%) were penta-drug refractory (≥2 immunomodulatory drugs, ≥2 proteasome inhibitors, and ≥1 anti-CD38 antibody) at study entry [12].

During the study, 48 patients (29.1%) had a COVID-19 infection overall (grade 3/4 in 35 patients [21.2%]) and 18 patients (10.9%) died from COVID-19 [9]. Four deaths from COVID-19 were considered by the investigators to be related to teclistamab treatment. Overall, supportive therapies (such as glucocorticoids, anti-infectives, hyperimmune plasma, and tocilizumab) were used to treat COVID-19 in 24.2% of patients [9], including antivirals (remdesivir) in 9.1%; Teclistamab dosing was interrupted in 29 of the 48 COVID-19 cases [9]. Prior to receiving the first teclistamab dose, 13 patients (7.9%) had received ≥1 COVID-19 vaccination, including one of the 18 patients who died of COVID-19 (who received two doses before starting teclistamab). As previously reported, during treatment with teclistamab, 99 patients (60.0%) received ≥1 COVID-19 vaccination dose on-study, including 13 of the 18 patients who died of COVID-19 (one dose, *n* = 3; two doses, *n* = 4; three doses, *n* = 3; four doses, *n* = 3 [the patient who was vaccinated pre-teclistamab received their third dose on-study]) [9]. Patients who had never been vaccinated were at an increased risk of infection with COVID-19 and tended to die of COVID-19 earlier during teclistamab treatment than those who had received ≥1 vaccination dose [9], with a greater proportion of deaths occurring in 2021 (5/5 versus 5/13, respectively) than 2022 (0/5 vs 8/13, respectively), likely reflecting the timing of broader global COVID-19 vaccine rollout.

After a median follow-up of 22.8 months in the overall study population, median PFS was 11.3 months (95% confidence interval [CI]: 8.8–16.4), median OS was 21.9 months (95% CI: 15.1–not estimable [NE]), and median DOR was 21.6 months (95% CI: 16.2–NE; Table 1 and Fig. 1). When censored for COVID-19 deaths, outcomes were prolonged: median PFS was 15.1 months (95% CI: 9.9–22.8), median OS was 28.3 months (95% CI: 21.9–NE), and median DOR was 26.7 months (95% CI: 21.6–NE; Table 1 and Fig. 1). The same trends toward prolonged survival and DOR were observed across the evaluated subgroups following censoring for COVID-19 deaths (Table 1 and Supplemental Fig. 1). This included the subgroup of patients who had received ≤3 prior lines of therapy, in which median PFS and OS also remained numerically higher than the overall study population regardless of the inclusion of COVID-19 deaths. These patients were earlier in their treatment journey and may therefore have had more favorable baseline immune fitness, which is important in achieving a response to teclistamab [13].

**Table 1:** Outcomes with teclistamab in the RP2D cohort of MajesTEC-1 in the overall study analysis and when censored for COVID-19 deaths.

Outcome	Population	Overall analysis (uncensored)^a^	Censored for COVID-19 deaths^a^
Median PFS, months (95% CI)	All patients (*N* = 165)^b^	11.3 (8.8–16.4)	15.1 (9.9–22.8)
	Patients with ≥CR (*n* = 75)^c^	26.9 (22.8–NE)	NE (26.9–NE)
	≤3 prior lines of therapy (*n* = 43)^d^	18.1 (13.8–26.9)	26.9 (13.8–NE)
	>3 prior lines of therapy (*n* = 122)^e^	9.7 (6.4–13.1)	10.8 (7.1–21.0)
	Phase 2 efficacy population (*n* = 110)^f^	10.8 (7.4–16.4)	13.8 (8.8–NE)
Median OS, months (95% CI)	All patients (*N* = 165)^b^	21.9 (15.1–NE)	28.3 (21.9–NE)
	Patients with ≥CR (*n* = 75)^c^	NE (NE–NE)	NE (NE–NE)
	≤3 prior lines of therapy (*n* = 43)^d^	25.9 (18.3–NE)	NE (21.7–NE)
	>3 prior lines of therapy (*n* = 122)^e^	17.7 (12.2–NE)	28.3 (16.0–NE)
	Phase 2 efficacy population (*n* = 110)^f^	21.7 (12.7–NE)	NE (21.7–NE)
Median DOR, months (95% CI)	All patients (*N* = 104)^b^	21.6 (16.2–NE)	26.7 (21.6–NE)
	Patients with ≥CR (*n* = 75)^c^	26.7 (21.6–NE)	NE (26.7–NE)
	≤3 prior lines of therapy (*n* = 32)^d^	21.1 (14.0–NE)	26.7 (15.9–NE)
	>3 prior lines of therapy (*n* = 72)^e^	NE (14.9–NE)	NE (20.1–NE)
	Phase 2 efficacy population (*n* = 68)^f^	21.6 (14.9–NE)	NE (21.6–NE)

*CI* confidence interval, *CR* complete response, *DOR* duration of response, *NE* non-estimable, *OS* overall survival, *PFS* progression-free survival, *RP2D* recommended phase 2 dose.

^a^Estimated median follow-up was 22.8 months.

^b^18 patients died from COVID-19 in the RP2D cohort of MajesTEC-1 (*N* = 165); 17 were censored in the PFS analysis, 18 in the OS analysis, and 13 in the DOR analysis.

^c^7 patients were censored in the PFS, OS, and DOR analyses.

^d^5 patients were censored in the PFS, OS, and DOR analyses.

^e^12 patients were censored in the PFS analysis, 14 in the OS analysis, and 8 in the DOR analysis.

^f^13 patients were censored in the PFS analysis, 15 in the OS analysis, and 9 in the DOR analysis.

**Fig. 1:**
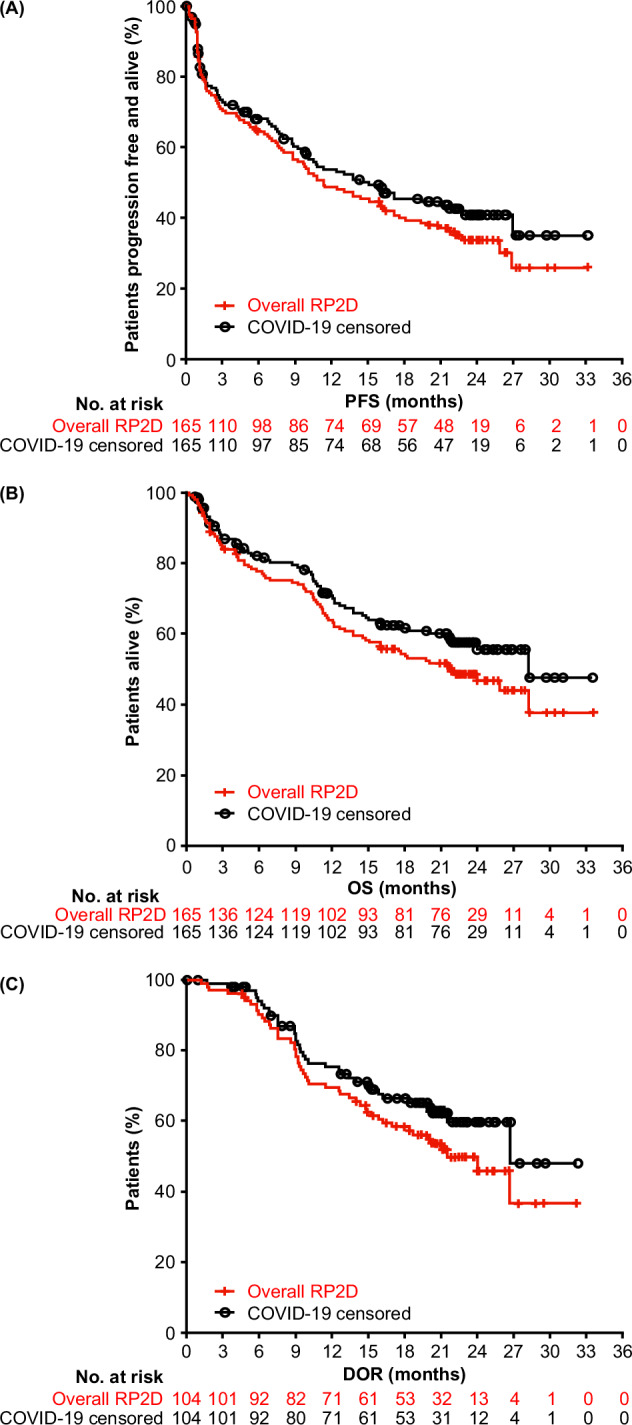
Outcomes with teclistamab in the RP2D cohort of MajesTEC-1 in the overall study analysis and when censored for COVID-19 deaths.^a,b^ **A** PFS, (**B**) OS, and (**C**) DOR. *DOR* duration of response, OS overall survival, PFS progression-free survival, RP2D recommended phase 2 dose. ^a^Estimated median follow-up was 22.8 months. ^b^18 patients died from COVID-19 in the RP2D cohort of MajesTEC-1 (*N* = 165); 17 were censored in the PFS analysis, 13 in the DOR analysis, and 18 in the OS analysis.

Our findings underscore the critical importance of appropriate infection prevention, monitoring, and management in optimizing outcomes for patients with RRMM treated with teclistamab. Clinical practice is now guided by published consensus guidelines [1] together with strong expert alignment [14] on managing infections (including COVID-19) in MM generally and with BCMA-directed therapy (such as bispecific antibodies) specifically. Importantly, this includes the routine use of immunoglobulin replacement, which was variable across institutions involved in MajesTEC-1 but is now widely recommended as an essential measure to mitigate infection risk in patients receiving BCMA-directed therapy, especially those with hypogammaglobulinemia [1, 9, 14]. Immunoglobulin replacement may also be particularly useful due to the quantity of COVID-19 antibodies it now contains [14] compared with immunoglobulin preparations during the early part of the pandemic, which had lower protective antibody titers. Similarly, vaccination against COVID-19 is also strongly recommended for all patients with MM in published guidance [14, 15], including for those with hypogammaglobulinemia, which has also been linked to poor vaccine response in MM [15]—again, underlining the importance of immunoglobulin replacement in this already immunocompromised population. It is important to note that patients with MM may have suboptimal antibody responses to vaccination, and emerging data suggest that concurrent or recent BCMA-directed therapy may contribute to poor response [15], indicating that even when available, COVID-19 vaccination during MajesTEC-1 may not have provided optimal protection.

As outlined above, little was known about the impact of COVID-19 infection on heavily pretreated patients with RRMM at the onset of the COVID-19 pandemic, with no published consensus guidelines on infections with BCMA-directed bispecific antibodies available during MajesTEC-1 enrollment. The rates of infection, including COVID-19, and efficacy outcomes seen in MajesTEC-1 should be viewed in the context of the timing of the study, with future studies likely to provide a more representative clinical picture for teclistamab [2]. More than 12,000 patients worldwide have now been treated with teclistamab since the first patient was dosed in clinical trials; real-world data with teclistamab suggest that the worldwide decrease in rates of COVID-19 infection and death [8] is being reflected in current clinical practice, and additional data from this setting will guide further understanding of outcomes in the prevailing period of both innate and vaccine-induced immunity among patients with MM. In addition, further evidence from randomized, controlled studies would be valuable in evaluating the true risk of infection across this class of drugs.

## Supplementary information

Supporting Information

## Data Availability

The data sharing policy of Janssen Pharmaceutical Companies of Johnson & Johnson is available at https://www.janssen.com/clinical-trials/transparency. As noted on this site, requests for access to the study data can be submitted through Yale Open Data Access (YODA) Project site at http://yoda.yale.edu.

## References

[1] Raje N, Anaissie E, Kumar S, Lonial S, Martin T, Gertz M, et al. Consensus guidelines and recommendations for infection prevention in multiple myeloma: a report from the International Myeloma Working Group. Lancet Haematol. 2022;9:e143–161. 35114152 10.1016/S2352-3026(21)00283-0

[2] Rubinstein S, Derman B. Infection rates are high across the multiple myeloma continuum, not just with bispecific antibodies. Eur J Cancer. 2023;189:112926. 37307686 10.1016/j.ejca.2023.05.014

[3] Martinez-Lopez J, Hernandez-Ibarburu G, Alonso R, Sanchez-Pina J, Zamanillo I, Lopez-Munoz N, et al. Impact of COVID-19 in patients with multiple myeloma based on a global data network. Blood Cancer J. 2021;11:198. 34893583 10.1038/s41408-021-00588-z PMC8661359

[4] Al-Kuraishy H, Al-Gareeb A, Mohammed A, Alexiou A, Papadakis M, El-Saber Bathia G. The potential link between Covid-19 and multiple myeloma: a new saga. Immun Inflamm Dis. 2022;10:e701. 36444620 10.1002/iid3.701 PMC9673426

[5] Chari A, Samur K, Martinez-Lopez J, Cook G, Biran N, Yong K, et al. Clinical features associated with COVID-19 outcome in multiple myeloma: first results from the International Myeloma Society data set. Blood. 2020;136:3033–40. 33367546 10.1182/blood.2020008150 PMC7759145

[6] Curigliano G, Banejee S, Cervantes A, Garassino M, Garriso P, Girard N, et al. Managing cancer patients during the COVID-19 pandemic: an ESMO multidisciplinary expert consensus. Ann Oncol. 2020;31:1320–35. 32745693 10.1016/j.annonc.2020.07.010 PMC7836806

[7] Hungria V, Garnica M, de Queiroz Crusoé E, de Magalhaes Filho R, Martinez G, Bittencourt R, et al. Managing patients with multiple myeloma during the COVID-19 pandemic: recommendations from an expert panel – ABHH Monoclonal Gammopathies Committee. Hematol Transfus Cell Ther. 2020;42:200–5. 32405620 10.1016/j.htct.2020.05.001 PMC7218372

[8] Mathieu E, Ritchie H, Rodés-Guirao L, Appel C, Giattino C, Hasell J, et al. Coronavirus pandemic (COVID-19). 2020. https://ourworldindata.org/coronavirus.

[9] Nooka AK, Rodriguez C, Mateos M-V, Manier S, Chastain K, Banerjee A, et al. Incidence, timing, and management of infections in patients receiving teclistamab for the treatment of relapsed/refractory multiple myeloma in the MajesTEC-1 study. Cancer. 2024;130:886–900. 37960969 10.1002/cncr.35107

[10] Tregoning J, Flight K, Higham S, Wang Z, Pierce B. Progress of the COVID-19 vaccine effort: viruses, vaccines and variants versus efficacy, effectiveness and escape. Nat Rev Immunol. 2021;21:626–36. 34373623 10.1038/s41577-021-00592-1 PMC8351583

[11] US Food and Drug Administration. Coronavirus (COVID-19) update: FDA authorizes first oral antiviral for treatment of COVID-19. 2021. https://www.fda.gov/news-events/press-announcements/coronavirus-covid-19-update-fda-authorizes-first-oral-antiviral-treatment-covid-19.

[12] Moreau P, Garfall AL, van de Donk NWCJ, Nahi H, San-Miguel JF, Oriel A, et al. Teclistamab in relapsed or refractory multiple myeloma. N Engl J Med. 2022;387:495–505. 35661166 10.1056/NEJMoa2203478 PMC10587778

[13] Cortes-Selva D, Casneuf T, Vishwamitra D, Stein S, Perova T, Skerget S, et al. Teclistamab, a B-cell maturation antigen × CD3 bispecific antibody, in patients with relapsed/refractory multiple myeloma: correlative analyses from MajesTEC‑1. Blood. 2022;140:241–3.

[14] Mohan M, Chakraborty R, Bal S, Nellore A, Baljevic M, D’Souza A, et al. Recommendations on prevention of infections during chimeric antigen receptor T-cell and bispecific antibody therapy in multiple myeloma. Br J Haematol. 2023;203:736–46. 37287117 10.1111/bjh.18909 PMC10700672

[15] Ludwig H, Sonneveld P, Facon T, San-Miguel J, Avet-Loiseau H, Mohty M, et al. COVID-19 vaccination in patients with multiple myeloma: a consensus of the European Myeloma Network. Lancet Haematol. 2021;8:e934–e946. 34756169 10.1016/S2352-3026(21)00278-7 PMC8553271

[16] Moreau P, Mateos M-V, Gonzalez Garcia ME, Einsele H, De Stefano V, Karlin L, et al. Comparative effectiveness of teclistamab versus real-world physician’s choice of therapy in LocoMMotion and MoMMent in triple-class exposed relapsed/refractory multiple myeloma. Adv Ther. 2024;41:696–715. 38110653 10.1007/s12325-023-02738-0 PMC10838813

