# Supplementary material for: Impact of COVID-19 on outcomes with teclistamab in patients with relapsed/refractory multiple myeloma in the phase 1/2 MajesTEC-1 study
Source: Blood Cancer J. 2024 Oct 21;14(1):186. doi: 10.1038/s41408-024-01160-1 (PMC11494165; doi:10.1038/s41408-024-01160-1)
Supplement: Supplementary file 1 — Supporting Information [file 41408_2024_1160_MOESM1_ESM.pdf]

Supplemental Figure 1. (A) PFS, (B) OS, and (C) DOR with teclistamab in the RP2D cohort of MajesTEC-1 in patients with ≥CR, by number of prior lines of therapy (≤3 or >3), and in the phase 2 efficacy population of MajesTEC-1[a] in the overall study analysis and when censored for COVID-19 deaths.[b,c]

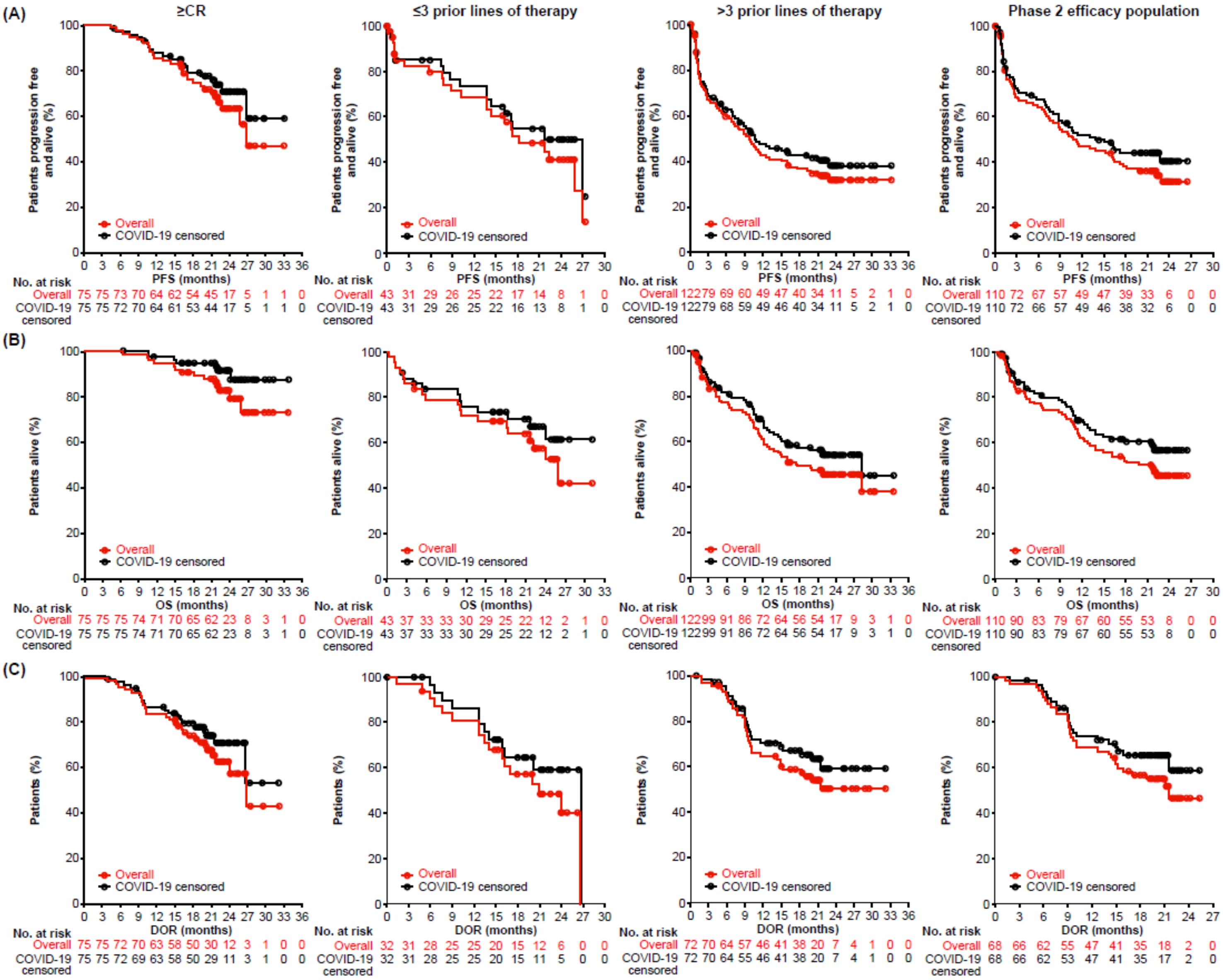

CR complete response, *DOR* duration of response, *OS* overall survival, *PFS* progression-free survival, *RP2D* recommended phase 2 dose.

[a]The phase 2 efficacy population included 110 patients enrolled on or before March 18, 2021.

[b]Estimated median follow-up was 22.8 months.

[c]In the subgroup of patients with ≥CR, 7 patients were censored in the PFS, OS, and DOR analyses. In patients who had received ≤3 prior lines of therapy, 5 patients were censored in the PFS, OS, and DOR analyses. In patients who had received >3 prior lines of therapy, 12 patients were censored in the PFS analysis, 14 in the OS analysis, and 8 in the DOR analysis. In the phase 2 efficacy population, 13 patients were censored in the PFS analysis, 15 in the OS analysis, and 9 in the DOR analysis.
